# Sexually Different Parental Inputs in Pheasant-tailed Jacanas and the Correlates with Brood Success

**DOI:** 10.1093/iob/obaf035

**Published:** 2025-09-04

**Authors:** Y-M Kuo, Y-F Lee, B-Y Chuang, Y-J Kuo, H-C Hsu, Y-Y Chiang, Y-L Tai, S-L Chang, C-Y Lin, Y-J Huang, W-C Lee

**Affiliations:** Department of Life Sciences, National Cheng Kung University, Tainan 70101, Taiwan; Department of Life Sciences, National Cheng Kung University, Tainan 70101, Taiwan; Department of Life Sciences, National Cheng Kung University, Tainan 70101, Taiwan; Department of Life Sciences, National Cheng Kung University, Tainan 70101, Taiwan; Department of Life Sciences, National Cheng Kung University, Tainan 70101, Taiwan; Department of Life Sciences, National Cheng Kung University, Tainan 70101, Taiwan; Department of Life Sciences, National Cheng Kung University, Tainan 70101, Taiwan; Department of Life Sciences, National Cheng Kung University, Tainan 70101, Taiwan; Department of Life Sciences, National Cheng Kung University, Tainan 70101, Taiwan; Department of Life Sciences, National Cheng Kung University, Tainan 70101, Taiwan; Jacana Ecological Education Center, Tainan 72099, Taiwan; Jacana Ecological Education Center, Tainan 72099, Taiwan

## Abstract

Parental care and territoriality are crucial components for the success of avian reproduction. Biparental care with female-biased efforts prevails in avian species, whereas breeding territories in most birds are male- or bisexual-defended. In social-polyandrous birds, however, females trade parental care for mating through sex-role reversal. On the other hand, managing multiple broods or mating events exposes females to physiological/environmental constraints of energetic-nutritional demands, which in turn may result in variations in egg mass and subsequent egg fates. This study assessed sexual differences in parental efforts, including territoriality, time allocation of parental behaviors, and egg-laying (reflected by egg mass) in sex-role-reversed pheasant-tailed jacanas, *Hydrophasianus chirurgus*, and their relationships with brood success. Females monopolized small ponds but shared larger ones with female neighbors by holding larger territories. In contrast, male territories were within those of their mates, the size was not affected by the presence of male neighbors, and was associated with the total hatchlings and fledglings obtained through multiple clutches. The time allocated in parental behaviors differed between the sexes and across the pre-laying, incubation, and post-hatching stages. The breeding duration, territory size, female breeding order, and male mating order, however, had no effects on parental time allocation. While male time spent chick-attending was positively correlated with brood success, preening negatively correlated with the fledging rate, other behaviors had no effects on reproductive outputs. The egg mass varied slightly, but showed no effect of year, nor the season of laying date until late August. The fourth egg in a clutch was lighter and, among clutches, the egg mass tended to be greater in later clutches and clutches from polyandrous females. We found positive correlations between mean egg mass and the numbers of hatchlings and fledglings gained per clutch. Our results suggest a substantial pre-laying parental input through egg production in polyandrous females. Brood success, however, appears to be determined by the combined effects of multiple factors, including male devotion and environmental conditions.

## Chinese abstract

親代撫育及領域性是促進鳥類生殖成功的關鍵因素。以母鳥為主的雙親撫育盛行於多數鳥類, 生殖期領域則多半由雄鳥或雙親共同守護。然而, 在社會性一妻多夫的鳥種中, 雌鳥卻透過性別角色翻轉以親代撫育換取配對機會;另一方面, 這些雌鳥在多次產卵或交配下的能量與養分需求, 仍面臨來自生理或環境的限制, 進而可能造成雌鳥產卵重量的變異並影響子代未來命運。本研究針對具有性別角色翻轉特徵的雉尾水雉 (*Hydrophasianus chirurgus*), 評估親鳥在育幼投入, 包括領域性、育幼行為的時間分配 (time allocation) 、及產蛋上的性別差異, 並分析其與生殖成功間的關聯。結果發現雉尾水雉雌鳥往往獨占小型池塘, 在較大水池中才與其他鄰近雌水雉分享水域, 但傾向佔據較大領域面積。相反地, 雄水雉的領域完全位在其雌性配偶的領域內, 領域大小不受是否有鄰近雄鳥存在的影響, 但正向關聯到孵化與存活的雛鳥數。親鳥撫育行為的時間分配有明顯性別間及產卵前、孵卵期間、與孵化後等階段間差異。然而, 生殖期程長度、領域面積大小、雌鳥生殖或雄鳥交配的先後順序對親鳥撫育行為的時間分配皆無影響。另一方面, 親鳥看顧雛鳥的時間長短與生殖成功呈正相關, 理羽所花時間長短與雛鳥存活率呈負相關, 但其他行為對水雉生殖產出皆無明顯效應。雉尾水雉的蛋重量與形值有些許變異, 但不具年間與產蛋期間的差異。雌鳥每窩蛋的第四顆一般較輕;各窩蛋之間, 則以較後期所產以及配偶數較多的雌鳥所產的蛋, 平均較重。每窩蛋的均重則和每窩平均孵化與存活的雛鳥數呈正相關。本研究結果顯示, 行一妻多夫的雌性雉尾水雉在生殖期間, 透過產蛋投入顯著的產卵前撫育成本;然而生殖成功本身, 則受到包含雄鳥撫育所投入程度及環境因素等多重因子的綜合影響。

## Introduction

Parental investment, in its broad sense, includes all parental expenditures (e.g., energy, time, or resources) stemming from parental behaviors or non-behavioral efforts that enhance the growth and survival of offspring but at a potential cost to the parents’ own fitness, including future mating success, fecundity, or survival (Trivers 1972; Clutton-Brock 1991; Kokko and Jennions 2012). In many species, parental care and investment differ between the sexes (Clutton-Brock 1991; Kokko and Jennions 2012), and these differences have profound connections with and effects on the intensity of sexual conflicts and selection, with the sex investing less predicted to be more eager in seeking for mating accesses and the sex investing more should be more discriminating (McNamara and Wolf 2015; Fritzsche et al. 2021).

In birds, parental care typically involves preparing rearing environments (e.g., nesting), egg production, brooding, and provisioning or attending post-hatching young (Clutton-Brock 1991). These activities, particularly brooding and rearing young, are considered energetically demanding (Ricklefs 1974; Nord and Williams 2015) and can be immunologically costly to the parents (Hanssen et al. 2005). Therefore, properly allocating available time among various behaviors or activities is important to the parents’ fitness (e.g., Weathers and Sullivan 1993; Tulp and Schekkerman 2006; Elliott et al. 2008). The time spent allocating among parental care and other activities usually reflects physiological tradeoffs and behavioral adjustments aimed at coping with changes associated with various spatiotemporal situations in the environment (e.g., seasonality) and life history stages (Tulp and Schekkerman 2006; Varpe 2017). As a result, it is expected to differ across species or even among individuals, depending on the type or extent of parental care required (Szekély and Cuthill 1999).

Unlike parental care contributing directly to rearing offspring, territoriality in birds serves multiple functions, including, but not limited to, mate attraction and pair bond maintenance (Brown 1969; Mentesana et al. 2020). Territoriality also plays a role in avian reproduction through acquiring and aiding the defense of nesting sites, access to resources, or both, which are closely associated with, or influential on, those direct forms of parental care (Brown 1969; Mentesana et al. 2020). Consequently, the size and quality of territory may affect the clutch size or other components of brood success, although also often through interactions with individual factors (Högstedt 1980; Goodburn 1991; Both and Visser 2000). This is evident in both terrestrial birds (e.g., Goodburn 1991; Przybylo et al. 2001; Zabala and Zuberogoitia 2014; Flockhart et al. 2016) and shorebirds (Ens et al. 1992; Parish and Coulson 1998; Yasué and Dearden 2008).

Social polyandry is a rare mating system, and in birds, except the altricial black coucals (*Centropus grillii*, Goymann et al. 2015), it is limited to certain charadriiform shorebirds that wander along shores or inland water bodies, such as jacanids, rostratulids, a few charadriids and scolopacids, and the terrestrial plains-wanderers *Pedionomus torquatus* and buttonquails (*Turnix* spp., Ligon 1999; Fritzsche et al. 2021). Most shorebird territories are male-defended (Szekély et al. 2024). Yet, socially polyandrous birds are typically associated with sex-role reversal in both morphology (e.g., larger-sized and brighter/more ornamented females than males) and behaviors (e.g., more aggressive and territorial females than males, but sole care of their precocious and nidifugous young by the male; Temrin and Tullberg 1995; Cockburn 2006). Nevertheless, how and to what extent sexual differences in parental care and territoriality affect the brood success of socially polyandrous birds remains unclear.

Even with sex-role reversal, egg production remains the principal form of female parental expenditure. In polyandrous birds, females can produce multiple broods by mating with one or more mates (e.g., Schamel et al. 2004; Lee et al. 2024), which is costly for females in terms of both energy and nutrition (Carey 1996). In some cases, females may strategically reduce their body reserves (i.e., body condition) to force males into caring for them (Szekély et al. 1996; Barta et al. 2002), which may impact their ability to produce eggs (Gladbach et al. 2010). In addition, physiological constraints or trade-offs may gradually drain females, thus leading to reduced outputs and reproductive gains over time (Varpe 2017). A reduced clutch size or egg size may help alleviate these energetic constraints. Indeed, the clutch size across shorebird species is typically four (Lengyel et al. 2009), the socially polyandrous species tend to produce smaller-sized eggs (Liker et al. 2001), although this may be also subject to female-biased sexual size dimorphism (Lislevand and Thomas 2006).

Alternatively, but not mutually exclusively, social-polyandrous birds may benefit from adaptive intra-clutch variation in egg size, which can shorten the interclutch duration and enhance offspring diversity (e.g., differences in size and the exploratory or locomotive ability), as a bet-hedging strategy when coping with environmental uncertainty (Laaksonen 2004). This is similar to that ubiquitously observed in altricial and some semi-precocial birds through hatching asynchrony (Stenning 1996; Boseman 2014). Interclutch variation appears greater than intra-clutch variation in avian egg mass (Christians 2002), yet, both sources of variation in egg size has not been well studied for most birds (Maddox and Weatherhead 2008), particularly in social-polyandrous species (Eberhart-Hertel et al. 2023), including jacanas (Jacanidae) in the tropical areas (Osborne and Bourne 1977; Fazili et al 2013).

Accordingly, the present study examined the sexual differences in parental efforts and the brood success of socially polyandrous pheasant-tailed jacanas *Hydrophasianus chirgus*, which show intensive intrasexual territorial defense and agonistic interactions (Thong-aree et al. 1995; Fresneau et al. 2021), with a focus on the territorial size and time allocation of both sexes and the egg mass variation. We assessed the sexual differences in jacana territory and predicted that the presence of same-sex neighboring jacanas within the same waterbodies is associated with larger territory sizes and higher reproductive outputs of the respective sex. Second, we examined sexual differences in parental time allocation and predicted that the proportion of time spent on brooding and chick-caring is positively correlated with brood success. Third, we assessed the egg mass variation within a clutch and across clutches laid over the breeding season, at different laying order, and among females that laid varying number of clutches and eggs. We predicted that jacana eggs exhibit within clutch variation, with later-laid eggs being lighter to compensate for an asynchronous hatching strategy (Laaksonen 2004), and inter-clutch variation, which may reflect the reproductive advantage of polyandry (Lee et al. 2024).

## Methods

### Study animals and area

Pheasant-tailed jacanas (*H. chirgus*) are widely distributed across the Indomalayan region between Pakistan and the Philippines. In Taiwan, their distribution is limited to a few discrete locations within or near Tainan, where they are under legal protection owing to historical population declines (Lee et al. 2024). We conducted fieldwork in the Jacana Ecological Education Park (ca. 15 ha, hereafter referred to as the JEEP) in Guantian (23°11′04.2″N, 120°18′47.2″E, 35 m above sea level), Tainan, southern Taiwan. Guantian experiences mild tropical weather, with mean monthly temperatures ranging from approximately 18°C in January to ca. 30°C in July. This area receives annual precipitation of approximately 1700 mm, mainly concentrated in the plum rain-typhoon season, which extends from May to September (Central Weather Bureau 1981–2024 data). Established from abandoned and then restored crop fields, the JEEP comprises about a dozen ponds of different sizes (mean: 0.79 ± 0.21 ha, range: 0.03–1.81), shapes, and proximities, forming either continuous or some separate waterbodies divided by natural ridges, and planted with various species of aquatic vegetations that are suitable for jacana nesting and feeding (Lee et al. 2024).

### Data collection

Due to the critical status of pheasant-tailed jacanas in Taiwan, data collection was limited mostly to observational data in the field to avoid disturbing the birds and their clutches. Between 2019 and 2021, we conducted near-daily monitoring and observation of pheasant-tailed jacanas in the pond sites of the JEEP throughout the breeding season, defined as the laying date of the first egg to the time the last clutch fledged (generally from early April to late September; Lee et al. 2024). We used binoculars (Zeiss 10 × 30 Conquest Compact T*, Germany), telescopes (Kowa Prominar with TE-11WZ 25–60×/30–70 × eyepiece, Japan), stopwatches, and counters for our observations. We always positioned ourselves behind tree branches, underbrush, bushes, or bird blinds located at a strategic distance from the ponds to minimize disturbance to the birds. We conducted banding attempts only in the post-breeding season toward mid or late fall, and the permissible capture was restricted (Lee et al. 2024).

#### Breeding territory and behavioral time allocation

Each day, depending on the number of jacanas present as the season progressed, 1–3 observers visited the pond sites. Searching and observation started at sunrise and lasted until sunset. We adopted the focal sampling (Martin and Bateson 2007) to target prior-selected male or female jacanas residing in each designated pond, one bout for one bird and by one observer, and focused on breeding-related behaviors, including courtship, copulation, nesting, brooding (including egg-shading by standing above the eggs), and chick-attending (including defense from actual or potential risks and sheltering from environmental hazards, e.g., the sun, heat, heavy rains, and predators), together with other behaviors, including foraging, vigilance, preening (including bathing), movement (walking or flying), resting, and agonistic behaviors (Fresneau et al. 2021; Chuang 2022). Nesting, brooding, and chick-attending were typical parental behaviors. Vigilance refers to head held high and looking around with neck extended and beak pointing above the horizontal line or in a particular direction. Agonistic behavior refers to fighting-related behaviors, including dispute, aggressive, and threat display to and/or attack other individuals with feet or wings (Tarboton 1992), often at or near territory borders (Chuang 2022). During each observation bout, we tracked the relative position of each targeted jacana within a pond continuously and recorded at 10 min intervals, with the aid of plants and man-made reference points that grew or previously set along the banks and within each pond. These positions were later marked on a map. Tracking observations on any male stopped once its clutch failed, but resumed for the males remaining in a female’s territory with further breeding interactions. Any new arrivals were noted, and if they became residents, further observation was conducted.

The territoriality of jacanas with the presence of agonistic behaviors during the breeding season, and their morphological/behavioral characteristics, rendered individual identification relatively straightforward and less difficult. Individual identification was aided by distinct feather features on the head (e.g., the size and shape of the stripe on the top) and body (the size and shape of the side patch on the closed wings). Each targeted bird was photographed (or filmed, whenever possible), sketched with color markings, serially numbered with a unique code according to the pond site at which it was observed. Within each pond, we identified individual birds according to their territory ranges, morphological features, and particular markings. We distinguished the sex of the targeted birds based on their behaviors (e.g., courtship, egg-laying) and relative differences in body size. We used the plastic-colored bands (Avian ID, Cornwall, England) applied to the jacanas during current or prior sampling, which, however, had a low re-sight rate (Lee et al. 2024). Female territories were estimated for 2 years, and those of males for 1 year.

Each observation bout lasted for at least 1 h before shifting to another target, either in the same pond or in a different pond. We alternated the time period of the day when each specific bird was followed and observed, so any bird would receive multiple and similar number of observation bouts over the 3 periods (morning: dawn to 1000 h, midday: 1000–1400 h, and afternoon: 1400 h to sunset). If a targeted bird took off or vanished from the sight field, we waited for at least 15 min for it to return or emerge before shifting to the next target. We recorded and timed the durations of all the observed behaviors, calculated their percentages for each bout, and calculated mean time allocation for each individual from multiple bouts of observations for each jacana, for subsequent analysis. All observers received prior practices and tests to ensure the within- and among-individual consistency of data recorded. Bouts lasting less than 30 min were excluded from later analyses, and we obtained a total of 1108 effective bouts of observations over 1111.3 h.

#### Abundance, clutch locality, and reproductive outputs

Two to three times each week, we used the scan sampling (Martin and Bateson 2007) to assess the entire study area across all the ponds at dawn. On these occasions, we counted all the jacanas present in each pond, checked the status of the already present clutches, recorded the localities of any new clutches, and counted the total number of adults engaged in breeding, together with the respective clutches, eggs laid, hatchlings gained, and fledglings sighted up to the 8th week after hatching (see Lee et al. 2024 for more details). We conducted the most intensive and thorough monitoring possible; however, we acknowledge that the absence of a genetic parentage assessment limits our inferences.

#### Egg mass and morphometrics

We measured a total of 540 eggs from 143 clutches. Mass and morphometric parameters were measured for each egg of each clutch located at the sites. We always conducted the measurements in the morning and when the parents were away from the clutch, and restricted our measuring process to less than 15–20 min to minimize disturbance. We weighed the eggs to the nearest 0.1 g using an electronic balance (JYB-500, Jin Yuan, Taiwan), and the length and the diameter of the widest point of the egg to the nearest 0.01 mm using a digital Vernier caliper (TMC, Taiwan), in the day immediately after the female laid her third or last egg of the clutch, and for some clutches in the last week of the incubation. We distinguished the laying order of the eggs present in a recently laid clutch by their colors and made a small mark on each egg using waterproof pens. Pheasant-tails’ eggs are laid fresh olive green, and then gradually turn dark green and eventually dark brown a couple of days after laying (Weng and Wang 1999, Fazili et al. 2013).

Bird eggs can lose weight gradually during incubation because of water vapor conductance through the shell (Mueller et al. 2022). The water loss rate may not be strictly linear, but tends to be steadier in precocial birds (Booth and Rahn 1990). Accordingly, we performed the second measurement 2–3 days after the first measurement to calculate the mean loss rate of egg mass via water evaporation over the first few days of laying. This mass loss rate was used to backward estimate the initial mass of eggs, by incorporating the actual hatching dates of these eggs observed in the field and the nearly consistent incubation period of 28 days (Weng and Wang 1999, Chuang 2022), particularly for a few cases (11 clutches, 40 eggs) that were found in the middle stage of incubation period and the initial egg mass was missed.

### Data analysis

We used the data recorded during the observations to verify each breeding group within a specific pond, including the territorial females and their mates, which were also territorial with generally recognizable boundaries. The focal sampling data and scanning survey data were used to obtain the active breeding duration of the males, where the length of this period was estimated as the time between the first egg-laying of the first clutch and the last egg-laying of the final clutch. We estimated the overall territory of each targeted jacana using the minimum convex polygon method (MCP; Powell 2000) by tracking the movement of the birds and sketching their locational maps. We chose this method because of its relative simplicity and flexibility and focused solely on the total area used. We acknowledged the limitation of this method in ignoring the tendencies of a jacana visiting different parts of a territory (Powell 2000), which was not the study focus. We marked the MCPs of jacanas on maps, and then scanned the maps and imported the maps using ImageJ 1.52, and the territory size of each jacana was calculated (Chuang 2022).

We classified breeding females as monandrous, bi-androus, or polyandrous for females with single, two, or multiple (three or more) male mates, and calculated their reproductive outputs accordingly by counting all the clutches made. Male reproductive outputs were similarly classified according to their association with monandry, bi-andry, or polyandry breeding groups. For males belonging to a polyandrous group, a note was made of their mating order. Reproductive outputs included the eggs produced, hatchlings, and fledglings gained from each identified clutch. We also calculated the hatching rate (number of chicks hatched out of total eggs), fledging rate (number of hatchlings surviving up to the 8th week out of total hatchlings), and overall brood success rates (number of hatchlings surviving up to the 8th week out of total eggs laid).

All the summary statistics of the data are presented as mean ± standard error (SE) or relative proportion (%) values, unless otherwise noted. We conducted the statistical tests using STATISTICA 12 (StatSoft, Tulsa, Oklahoma, USA) or SPSS 28.0 (IBM Chicago, IL, USA) for Windows 10, with an alpha value of 0.05. The data that deviated from the requirements of normality or homoscedasticity were logarithmic transformed, or arcsine or square-root transformed for proportional or count data, respectively, or a generalized linear model (GLZ) was adopted. Levene’s test was used to examine the homogeneity of variance, and Cohen’s *d*, the effect size, was used to quantify the magnitude of difference between means (Zar 2010).

We used a general linear model (GLM) to examine the variation of the female territory size from the sampling year, the extent of female polyandry (monandry, bi-andry, or polyandry), and the presence of female neighbors, with the area of separated water bodies as a continuous variable. This analysis was then applied to examine the variation of the male territory size within female territories due to the presence of male neighbors. We also assessed the effects of individual territory size, treated as a continuous variable, along with the presence of same-sex neighbors (and the extent of polyandry for females), on the reproductive outputs (i.e., numbers of hatchlings and fledglings, and hatching and fledging rates) of female and male jacanas, respectively. We performed the GLM to compare the time allocation of the prime parental behaviors of the female and male jacanas among the three breeding stages (pre-laying, incubation, and post-hatching), with the observers’ ID as a factor and observation bouts as a random variable. When factor effects were detected, we used Tukey’s honestly significant difference (HSD) tests to locate which means were significantly different. The difference in behavioral time allocation between the males and females was examined by a GLZ via a normal log link function to account for the non-normal distribution due to some behaviors rarely performed by a particular sex. We used the GLM to examine the effect of mating order, and breeding duration and territory size as continuous variables, on time allocation of major behaviors of females and males, respectively. We further adopted the general linear mixed model (GLMM) with individual clutches as a random factor to examine the variation in the egg mass and morphometrics. GLM was also used to assess the within-clutch effects of the laying order on the mean egg mass, and GLZ was used to assess the effects of female jacanas (the extent of polyandry, and the number of clutches and eggs laid) on the among-clutch variation in the egg mass, with Levene’s test assessing the variance among different groups. Finally, we used correlation, or quadratic polynomial regression (for curvilinear data), analyses to examine the relationships between the mean egg mass and the number of clutches and eggs laid, the laying order of the clutches, and the number of hatchlings and fledglings gained, respectively.

## Results

### Breeding territories

The females held a mean territory of 0.32 ± 0.036 ha (range: 0.001–1.380; CV = 84.09; *n* = 57) during the breeding season. We found no effects of the sampling year (GLM; *F*_1, 48 _= 1.26, *P* = 0.267) or the extent of polyandry (*F*_2, 48 _= 0.95, *P* = 0.392) on the female territory sizes. While single females generally monopolized smaller ponds (<0.4 ha), two or more females shared larger ponds (*F*_1, 48 _= 17.86, *P* < 0.001) for increased territories of varying extents of overlap, although usually not during the same period, and these females tended to hold larger territories than those without neighbors (*F*_1, 48 _= 4.23, *P* < 0.05; HSD, *P* < 0.01; Table 1). The male territories (0.14 ± 0.015 ha, range: 0.001–0.532, CV = 79.31, *n* = 51) were exclusively located within the territory of their female mates and generally less than half of the female territories in size. The male territory size did not differ between males with or without neighboring males (*F*_1, 49_ = 0.11, *P* = 0.749; Table 1). Within the territories of polyandrous females, however, the first mates had a larger territory (0.19 ± 0.033 ha, CV = 69.99, *n* = 16; *t* = 2.04, *P* < 0.05) than subsequent mates (0.11 ± 0.020 ha, CV = 74.43).

**Table 1.** Mean (±SE) territory size (ha) of female and male pheasant-tailed jacanas (*n* = number of territories and birds) with and without neighboring jacanas of the same sex in the JEEP

	Neighbors present	Neighbors absent	*d*
Female	0.442 ± 0.006 (29)^***^	0.196 ± 0.019 (28)	1.026
	(0.353–0.540; 73.31)	(0.100–0.294; 51.73)	
Male	0.14 ± 0.016 (43)	0.12 ± 0.045 (8)	0.194
	(0.105–0.171; 75.05)	(0.039–0.192; 110.84)	

The 95% confidence interval and coefficient of variation (CV, %) in parentheses are underneath each category. The asterisk symbols indicate a significant difference in the mean territory size between the jacanas with and without the same-sex proximate neighbors present. Cohen’s *d* indicates the effect size.

^***^ *P* < 0.001.

**Fig. 1.**
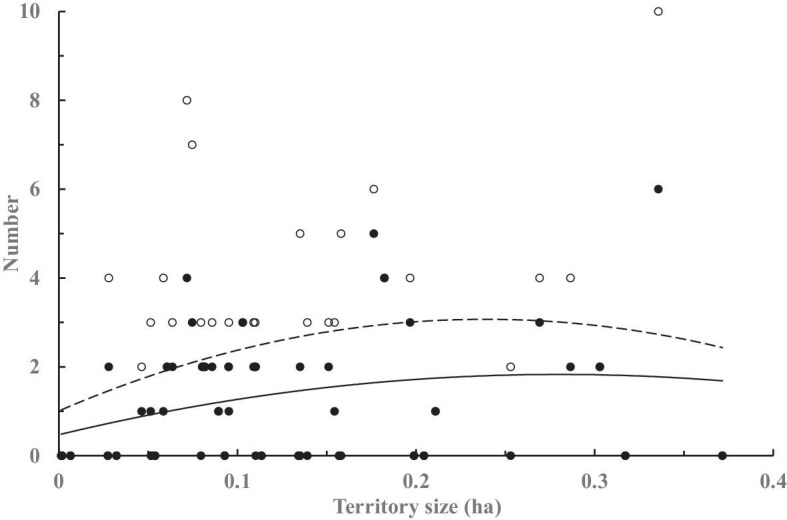
The number of hatchlings (∘) and fledglings (●) gained by the male pheasant-tailed jacanas (*n* = 51) of different territory sizes in the JEEP reserve.

The female territory size (Pillai–Bartlett’s trace value *V* = 0.57, *F*_10, 39_ = 5.14, *P* < 0.01), but not pond-sharing (*V* = 0.19, *F*_10, 39_ = 0.92, *P* = 0.52) or year (*V* = 0.24, *F*_10, 39_ = 1.24, *P* = 0.29), affected their reproductive outputs while interacting with the positive effect of polyandry (*V* = 0.67, *F*_20, 80_ = 2.02, *P* < 0.05; interaction: *V* = 0.37, *F*_10, 41_ = 2.46, *P* < 0.05). For males, we found a significant effect of the male territory size (*V* = 0.64, *F*_10, 21_ = 3.64, *P* < 0.01), but no effects of the presence of male neighbors (*V* = 0.34, *F*_10, 21_ = 1.10, *P* = 0.40), on the male reproductive outputs. The male territory showed a slightly curvilinear relationship with the total hatchlings (*r* = 0.26; GLZ: *χ*^2 ^= 21.05, *P* < 0.01) and fledglings obtained (*r* = 0.27; *χ*^2 ^= 9.28, *P* < 0.01) through multiple clutches, generally peaked at 0.1–0.2 ha with individual variation (Fig. 1), whereas male territory size had no relationships with either the hatching or fledging rates (*r* < 0.2).

### Time allocation between the sexes

**Fig. 2.**
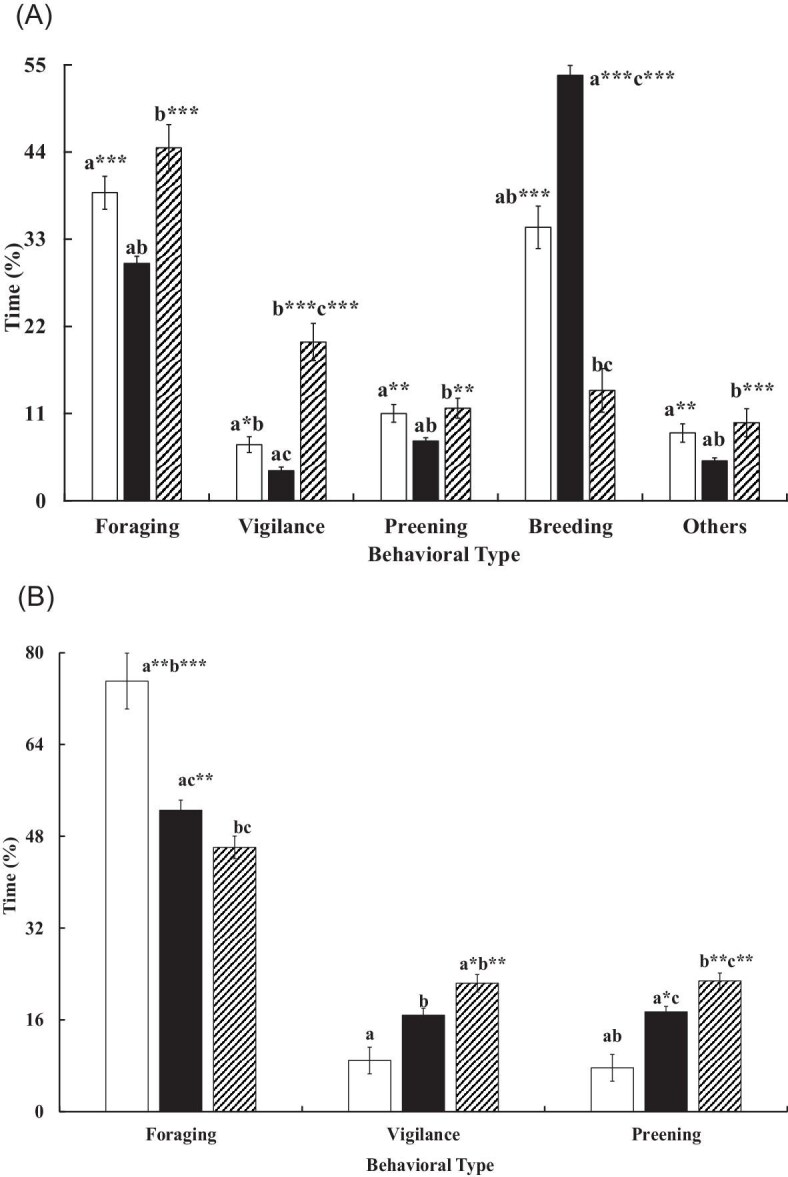
Mean (± SE) percentage of time spent by (A) male jacanas among major behaviors (foraging, breeding, vigilance, preening, and others) during pre-laying (**□**, *n* = 121**)**, brooding (■, *n* = 472), and chick-attending (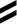, *n* = 91) periods, and (B) female jacanas among foraging, vigilance, and preening behaviors in pre-mating (**□**, *n* = 23), laying (■, *n* = 233), and post-laying (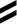, *n* = 178) periods at the bout level. A bar with a letter and an asterisk (*) indicates a significant difference in values between this bar and bars with the same letter within the same behavioral type. ^*^P < 0.05; ^**^  *P* < 0.01; ^****^P < 0.001. Breeding behavior for the males in the pre-laying period refers to courting, copulation, and nesting only, and to brooding and chick-attending specifically for each respective period. Others included movement, resting, and agonistic behaviors.

The breeding jacanas allocated time very differently between the sexes (Table 2). The females spent over half of their time foraging, followed by vigilance and preening, which collectivly accounted for over 88% of their daytime budget, but devoted no time to post-laying parental care. In contrast, the males allocated a significant portion of their time to breeding behaviors, particularly incubating (39.84%, *n* = 91 clutches), but spent lower portions of time compared to females in all other behaviors, including foraging (Table 2). Over observation bouts, the male time allocation varied among the pre-laying, incubating, and post-hatching stages (*V* = 0.28, *F*_8,1344_ = 27.05, *P* < 0.001), but not among observers (*V* = 0.04, *F*_24,2696_ = 1.06, *P* = 0.39). Breeding behaviors (courting-copulating-nesting, incubating, and chick attending) showed the largest among-stage margin, where incubating behavior dominated the other behaviors in the incubating stage (Fig. 2A). The female time allocation also differed among the pre-mating, laying, and post-laying stages (*V* = 0.07, *F*_6,826_ = 5.26, *P* < 0.001), but not among observers (*V* = 0.01, *F*_18,1242_ = 0.31, *P* = 0.998), with foraging showing the greatest deviation from other behaviors (Fig. 2B).

**Table 2.** Mean (±SE) percentage of time spent with 95% confidence interval and CV (%) in the parentheses, by female and male jacanas (*n* = number of birds, number of observation bouts) among various types of behaviors.

Behavior	Female (*n* = 26,424)	Male (*n* = 37,684)	*d*
Foraging^***^	50.67 ± 1.92 (46.86–54.48; 19.28)	33.84 ± 1.59 (30.65–37.03; 28.55)	1.732
Breeding^***^	1.82 ± 0.43 (0.93–2.71; 120.93)	43.55 ± 2.04 (40.38–46.71; 28.45)	4.690
Courting	0.18 ± 0.06 (0.05–0.31; 173.62)	0.51 ± 0.19 (0.22–0.81; 223.48)	0.396
Copulating	0.04 ± 0.03 (-0.02–0.10; 348.54)	0.10 ± 0.04 (0.03–0.17; 247.33)	0.294
Nesting	1.60 ± 0.43 (0.40–2.80; 135.39)	1.63 ± 0.58 (0.62–2.63; 218.6)	0.008
Brooding^***^	0.00 ± 0.00^1^ (0.00–0.001;—)	39.84 ± 2.00 (36.77–42.90; 30.48)	4.640
Chick-att^*^	0.00 ± 0.00^1^ (0.00–0.001;—)	1.47 ± 0.51 (0.68–2.26; 212.65)	0.665
Vigilance^***^	18.86 ± 1.18 (16.66–21.06; 31.95)	6.75 ± 0.87 (4.90–8.59; 78.61)	2.134
Preening^***^	19.07 ± 1.15 (17.24–20.90; 30.68)	8.74 ± 0.59 (7.21–10.27; 41.35)	2.125
Movement	3.06 ± 0.47 (1.72–4.39; 78.32)	2.98 ± 0.65 (1.86–4.10; 133.1)	0.024
Resting^**^	4.47 ± 0.60 (3.39–5.56; 68.14)	2.51 ± 0.42 (1.60–3.42; 101.42)	0.699
Agonistic	2.05 ± 0.45 (1.38–2.72; 112.08)	1.64 ± 0.19 (1.08–2.20; 68.99)	0.225

Breeding-related behaviors are indented. The asterisk symbols indicate a significant difference in the time allocation between sexes. Cohen’s *d* indicates the effect size.

^*^ *P* < 0.05; ^**^  *P* < 0.01; ^****^P < 0.001; ^1 ^< 0.0004.

**Fig. 3.**
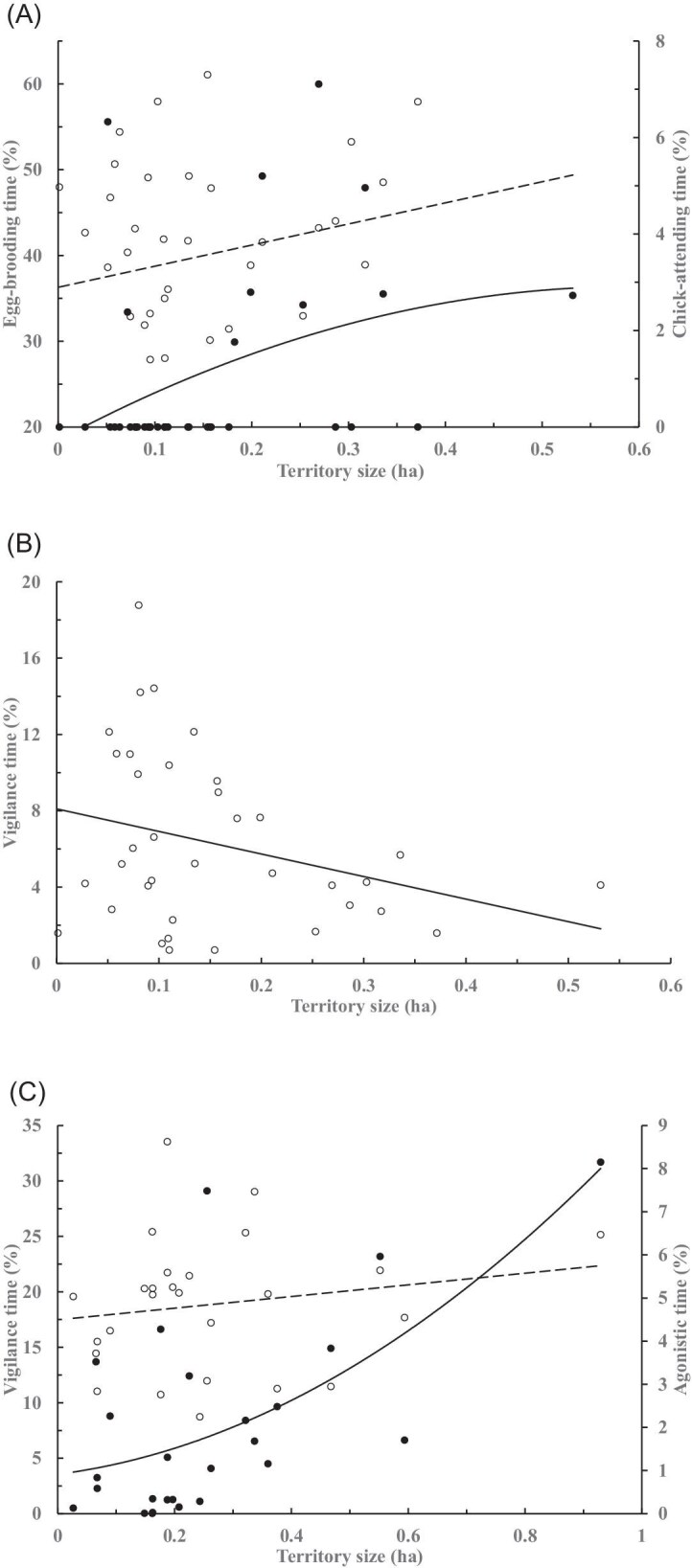
The time proportion spent by the male jacanas (*n* = 37) on (A) brooding (∘) and attending chicks (●), and (B) vigilance behaviors (∘), and that by the female jacanas (*n* = 26) on (C) vigilance (∘) and agonistic behavior (●), in relation to their territory sizes.

At the individual level, the time spent brooding by the males tended to increase (*r *= 0.19) but that of chick-attending varied slightly convexly (*r *= 0.38; Fig. 3A), whereas that of vigilance decreased (*r* = 0.30, Fig. 3B), with the territory size. In contrast, the time spent by the females increased sligthly on vigilance (*r *= 0.17), and concavely on agonistic behaviors (*r *= 0.69, *χ*^2^ = 13.44, *P* < 0.01), with the territory size (Fig. 3C). The among-jacana variation, however, was large. We found no effects of the breeding duration (female: *V* = 0.61, *F*_9, 13_ = 2.23, *P* = 0.092; male: *V* = 0.44, *F*_10, 24_ = 1.91, *P* = 0.094), the territory size (female: *V* = 0.63, *F*_9, 13_ = 2.47, *P* = 0.068; male: *V* = 0.29, *F*_10, 24_ = 0.97, *P* = 0.495), nor that of female breeding order within the same ponds (*V* = 0.96, *F*_18, 28_ = 1.44, *P* = 0.187) or male mating order of the same female mates (*V* = 0.12, *F*_10, 24_ = 0.33, *P* = 0.964), on the time allocation of major behaviors of females or males, respectively.

**Fig. 4.**
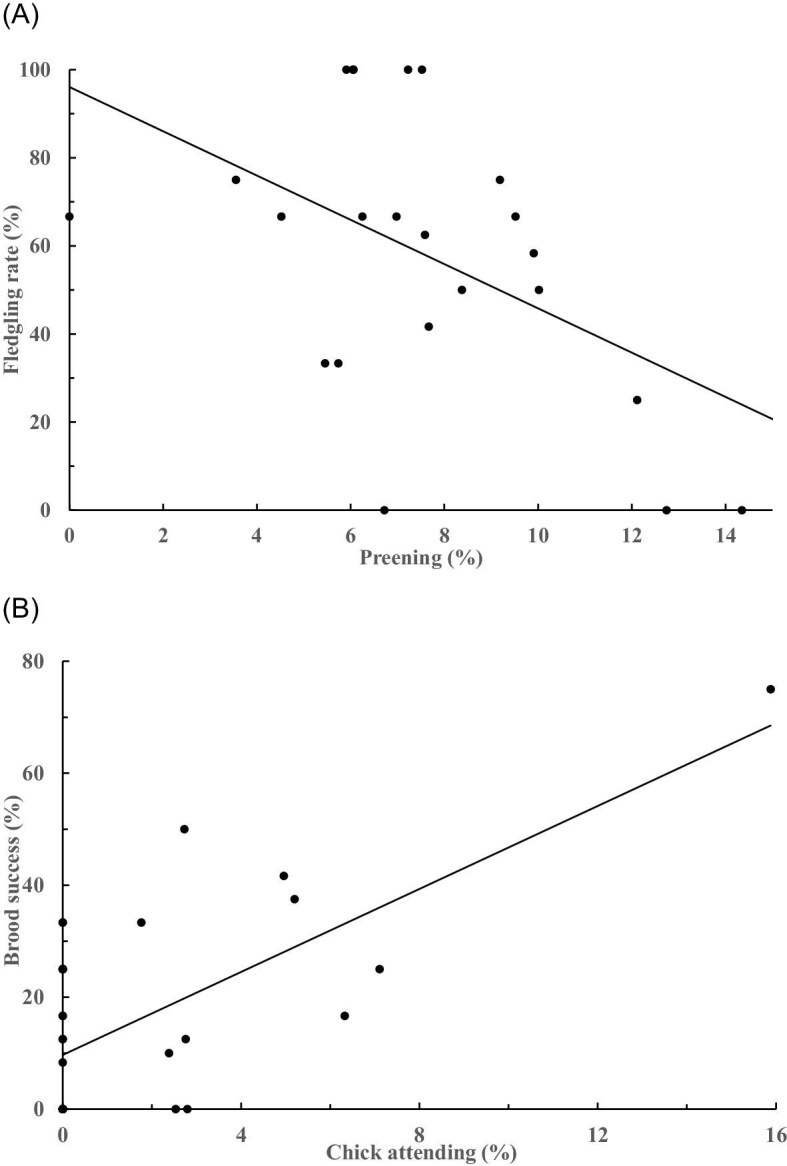
The fledging rate was (A) negatively, and the brood success was (B) positively, correlated to the time spent preening and chick-attending by the male jacanas (*n* = 37), respectively. The pattern of 4(B) held still significant without the outlier.

The time allocated among the females’ major behaviors (foraging, breeding, vigilance, and preening) had no relationship with any of the three reproductive outputs of females (hatching rate: *R^2^* = 0.21, *F*_4, 15_ = 1.02, *P* = 0.429; fledging rate: *R^2^* = 0.42, *F*_4, 15_ = 2.69, *P* = 0.072; brood success: *R^2^* = 0.17, *F*_4, 15_ = 0.74, *P* = 0.579). The males’ major behaviors moderately affected the fledging rates of males (*R^2^* = 0.37, *F*_4, 22_ = 3.21, *P* < 0.05), with preening (*V* = 0.338, *F*_3, 20_ = 3.40, *P* < 0.05) being negatively correlated with the fledging rates (*β* = −0.529, *t* = −2.75, *P* < 0.05; Fig. 4A), but showed no relationship with the hatching rates (*R^2^* = 0.23, *F*_4, 22_ = 1.65, *P* = 0.197) or brood success (*R^2^* = 0.08, *F*_4, 22_ = 0.46, *P* = 0.763) of males.

The three more eminent breeding behaviors of males (brooding, nesting, and chick attending) together also showed a moderate relationship with the brood success (*R^2^* = 0.41, *F*_3, 23_ = 5.25, *P* < 0.01), where chick attending (*V* = 0.507, *F*_3, 21_ = 7.19, *P* < 0.005) was positively correlated with the brood success (*β* = 0.616, *t* = 3.81, *P* < 0.05; Fig. 4B), whereas nesting (*V* = 0.04, *F*_3, 21_ = 0.29, *P* = 0.833) and brooding behaviors (*V* = 0.154, *F*_3, 21_ = 1.28, *P* = 0.308) had no effects. Similarly, these behaviors did not affect the hatching rate (*R^2^* = 0.22, *F*_3, 23_ = 2.13, *P* = 0.124) or fledging rate (*R^2^* = 0.12, *F*_3, 23_ = 0.997, *P* = 0.412).

### Egg mass variation

Females differed in the numbers of clutches (3.3 ± 0.25, range: 1–7) and eggs laid (12.0 ± 0.94, range: 3–28). The jacanas’ eggs varied only slightly in length (36.45 ± 0.083 mm, CV = 5.28), width (27.47 ± 0.086 mm, CV = 7.23; *n* = 540), and first-day mass (13.66 ± 0.058 g, CV = 9.83; *n* = 540). Most of this variation occurred among individual (GLMM: *F*_46,492_ = 9.58, *P* < 0.001), rather than between years (*F*_1,492_ = 1.76, *P* = 0.19).

**Fig. 5.**
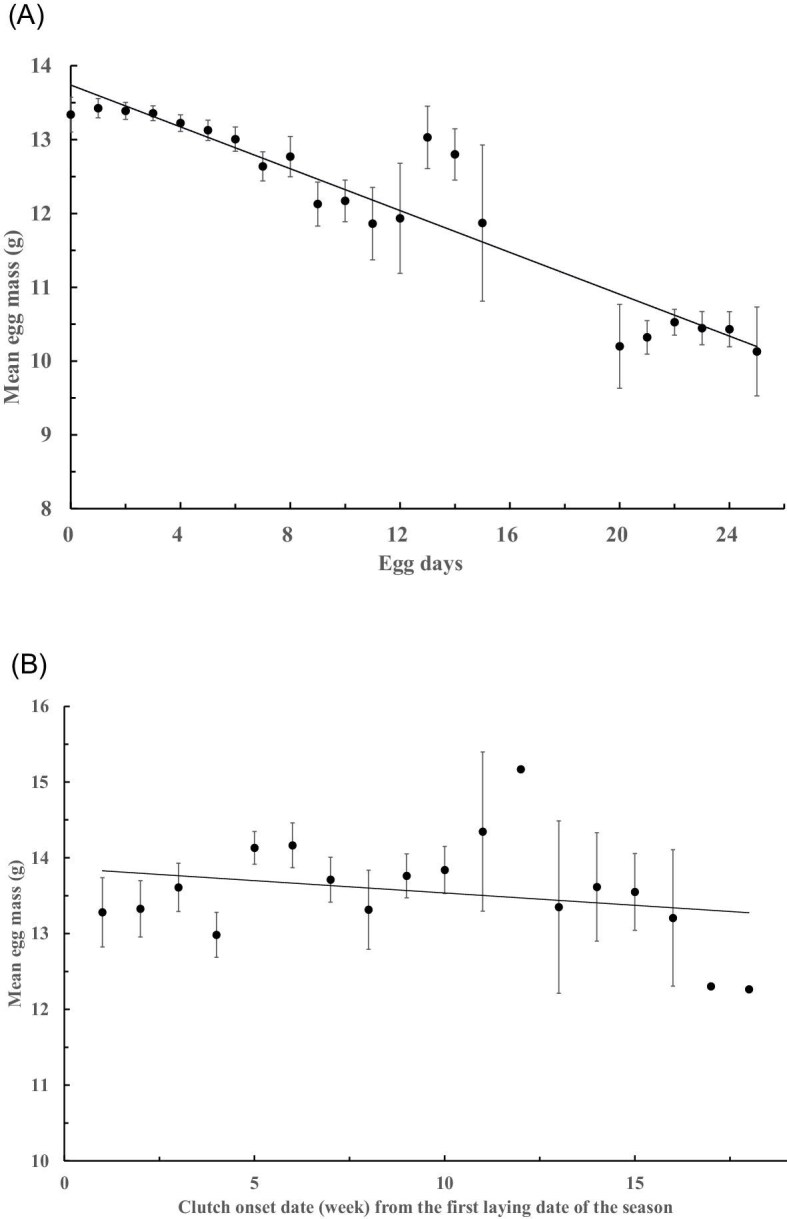
Mean (±SE) mass of eggs that were measured (A) at different days after laying decreased gradually (total *n* = 540 eggs), and (B) from clutches (total *n* = 139 clutches) laid at different times during the breeding season over approximately 18 weeks. Data for day 11 to day 20 presented in (A) were from the clutched that were measured in the middle stage of the incubation.

On average, the eggs lost a slightly higher proportion of its initial mass per day in the drier (2020: 0.012 ± 0.0004%, CV = 65.04, *n* = 313) than the wetter year (2021: 0.011 ± 0.0001%, CV = 18.33, *n* = 227; *P* < 0.05). Assuming this daily mass loss and incorporating with field measurements, the egg mass showed a gradual decline over the incubation period (*r* = 0.938, *P* < 0.05; Fig. 5A). However, the mean egg mass remained generally constant and showed a weak correlation with the initial laying date of the clutch (*r* = 0.252, *P* < 0.05), with slight fluctuations and increasing variation, until at least the 16th week of the season (approximately toward the end of August; Fig. 5B).

**Fig. 6.**
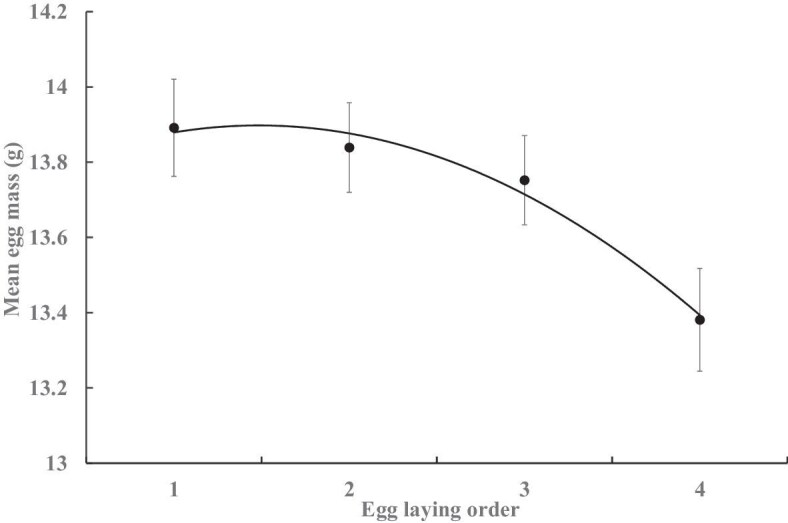
Mean (±SE) egg mass of different laying orders within the same clutch (*n* = 101,102, 102, and 91, respectively, for each order).

**Fig. 7.**
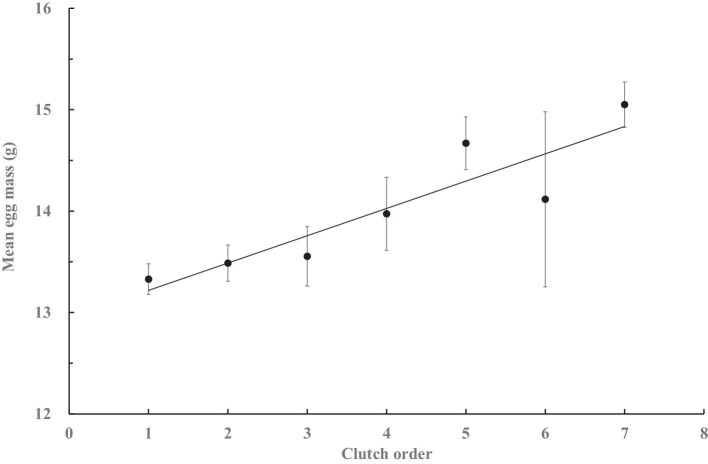
Mean (±SE) egg mass of clutches of different laying orders by female jacanas (*n* = 47, 30, 30, 18, 11, 4, and 3, respectively, for each order).

**Fig. 8.**
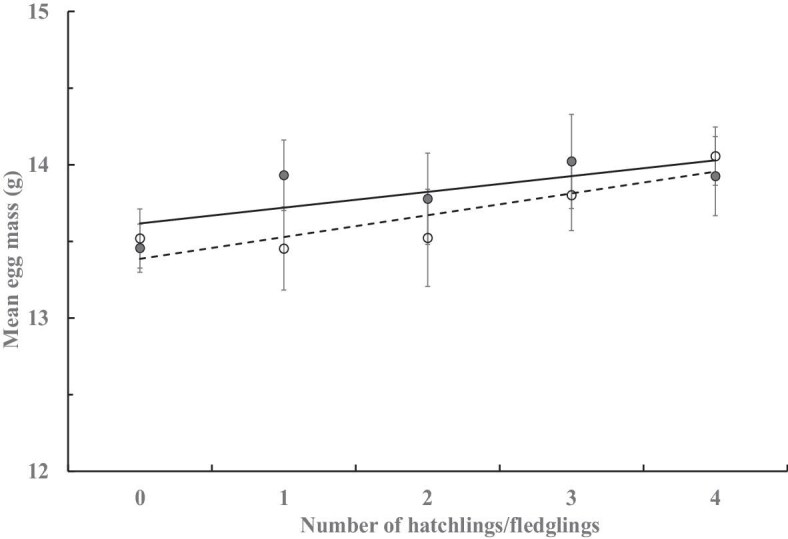
Mean (±SE) egg mass and its relationship with clutches with increased numbers of hatchlings (∘; *n* = 57, 19, 20, 20, and 27, respectively) and fledglings (●; *n* = 80, 21, 19, 11, and 12, respectively) gained per clutch.

Within a clutch, the egg mass varied among the laying order (GLM, *F*_3,348_ = 8.13, *P* < 0.001) under the random effect of female individuals (*F*_44,348_ = 10.13, *P* < 0.001), where egg mass tended to decline curvilinearly (*F*_1, 2_ = 9.20, *r* = 0.98, *P* = 0.094), with the 4th egg (*n* = 91) lighter than the first three eggs (*n* = 101 each, Tukey’s HSD, all *P* values <0.001; Levene’s test for homogeneity of variance, *F*_3,392_ = 0.37, *P* > 0.7; Fig. 6). Among clutches, the egg mass tended to increase in the clutches of later laying orders (*F*_1, 5_ = 22.88, *r* = 0.91, *P* < 0.005; Fig. 7). The mean egg mass did not vary among females laying different numbers of clutches (GLZ, *n* = 49; χ^2^* = *3.41, *P* = 0.471) or eggs (χ^2^* = *0.01, *P* = 0.905), but was higher in clutches of polyandrous females than that of monandrous and bi-androus females (χ^2^* = *6.18, *P* < 0.05; Table 3), with variance occurring both among groups of different clutch numbers (Levene’s test, *F*_4,44_ = 4.62, *P* < 0.005) and groups of mate numbers (*F*_2, 46_ = 5.70, *P* < 0.01). A greater mean egg mass was positively correlated with the number of hatchlings (*F*_1, 3_ = 10.96, *r* = 0.89, *P* < 0.05) and fledglings (*F*_1, 3_ = 3.44, *r* = 0.61, *P* < 0.05; Fig. 8) per clutch.

**Table 3.** Mean (± SE) egg mass and its 95% confidence interval, followed by coefficient of variation (CV, %) in the parentheses, of female jacanas (*n* = number of birds) in different mating patterns

Female	Monandry (19)	Bi-andry (20)	Polyandry (10)	*d*
Egg mass	13.50 ± 0.246^1^	13.27 ± 0.186^2^	14.32 ± 0.274^*1*2^	0.831^1^, 1.230^2^
	12.99–14.02 (6.28)	12.88–13.66 (6.05)	13.70–14.93 (7.95)	

The asterisk symbol and a letter indicate a significant difference in the mean egg mass between the clutches of polyandrous females and those from monandrous or bi-androus females, respectively, with the same letter. Cohen’s *d* indicates the effect size for the respective paired comparison of means.

^*^ *P* < 0.05.

## Discussion

The present study assessed sexual differences and variation in parental efforts, including territoriality, time allocation of parental behaviors, and egg-laying efforts reflected in egg mass in socially polyandrous and sex-role-reversed jacanas. Overall, we found a positive correlation of male parental care via chick attending, a slight curvilinear relationship of male territory size, and a positive effect of female territory size while through polyandry, with reproductive outputs of pheasant-tail jacanas. In egg mass, we found that the fourth egg in a clutch was generally lighter, whereas the mean egg mass in later clutches, and those from polyandrous females, was greater.

The size, or quality, or both, of territory may affect avian reproduction and the fitness of offspring through competition over food and space, which in turn influences their energy gains and safety from environmental hazards (Parish and Coulson 1998; Przybylo et al. 2001; Yasue and Dearden 2008; Flockhart et al. 2016; Mentesana et al. 2020). Shorebirds usually have male-defended or male-established bisexual-defended territories (Yasué and Dearden 2008; Szekély et al. 2024), and some sex-role reversed species are even nonterritorial (e.g., red-necked phalaropes *Phalaropus lobatus*, Schamel et al. 2004; red phalaropes *P. fulicarius*, Krietsch et al. 2022, Wilson’s phalarope *P. tricolor*, Szekély et al. 2024). However, both female and male jacanas establish and defend their respective territories (e.g., northern jacanas *Jacana spinosa*; Betts and Jenni 1991; bronze-winged jacanas *Metopidius indicus*, Butchart et al. 1999; and pheasant-tailed jacanas, Thong-aree et al. 1995, this study).

While the males in the present study held smaller territories exclusively within that of their mates, we found a curvilinear relationship of the size of male territories with the number of hatchlings and fledglings gained. This latter finding may also have contributed to the pattern observed for the female territory size through interaction with polyandry. In addition, the first mates of bi- or polyandrous females generally owned a larger territory, which is consistent with a previous finding, obtained at the same site, that the first mates gain a higher hatching success and number of hatchlings (Lee et al. 2024). On the other hand, the extent of polyandry or the presence of neighboring males had no effects on the respective territory size of females or males. In addition, we found no effects of pond-sharing by females or mate-sharing by males on the reproductive outputs of either sex, although the time spent on agonistic behavior tended to increase by the females in larger territories. The method used for estimating territories in our study may introduce some extent of spatial bias due to observational geometry, which applied to all territories estimated. Nevertheless, these results only partially support our prediction regarding jacanas’ territory size and reproductive outputs and suggest other factors than conspecific competition over food or space (Flockhart et al. 2016) for breeding opportunity (e.g., agonistic behaviors or egg-tossing by either males or females; Chen et al. 2008, Chuang 2022), may be also involved.

Territory size, depending on the availability of suitable habitats, food resources, and the density of con- and hetero-specific competitors, may vary as much as the territorial behavior (e.g., Lengyel et al. 2009, Flockhart et al. 2016). While high food abundance is often associated with reversed sex role in both shorebirds and black coucals (Safari and Goymann 2021; but also see Fresneau et al. 2024), the ponds in the present study experienced similar extents of aquatic vegetation (YF Lee, unpubl. data). In Thailand, brooding male pheasant-tailed jacanas typically defended a nesting site within a radius of 10–12 m (ca. 0.03 ha, Thong-aree et al. 1995); this behavior, however, was not observed in the present study. Meanwhile, in India, the territories of male bronze-winged jacanas were constrained by the presence of co-mates or neighboring males (Butchart et al. 1999), which was not observed in our study either. Nevertheless, Butchart et al. (1999) found that male dispersion affected female dispersion but was not related to resource dispersion, whereas female territories were positively correlated with the extent of polyandry, but unrelated to habitat quality. Unlike those studies in India and Thailand, which were conducted in single, large, and continuous waterbodies, the jacanas in our study occupied multiple ponds of various sizes, each separated by physical boundaries. This aligns with our finding that pond-sharing occurred only in larger ponds, where larger territories were more feasibly established.

Predation is known to be a significant biological factor affecting reproductive outputs of jacanas, alone or interacting with territory size, and has been documented to cause the loss of 80–90% of the clutches before hatching (e.g., bronze-winged jacanas, Butchart 2000; comb-crested jacanas *Irediparra gallinacean*, Mace 2000). The predators in our sites included at least birds of prey, egrets and herons, rat snakes (*Ptyas* spp.), stripe-necked turtles (*Mauremys sinensis*), and fishes such as black carp (*Mylopharyngodon piceus*) and striped snakehead (*Channa striata*) accounted for over one-third of the hatchling mortality (Lee et al. 2024, YF Lee, unpubl. data). The even lower number of fledglings than hatchlings gained may have resulted from additional and increased ease of predation on precocious and nidifugous young in larger territories held by less attentive males. This is also evident by the results of male time allocated to chick-attending and vigilance over increased territory size.

The behavioral time allocation in pheasant-tailed jacanas differed between the sexes, where males took sole responsibility for post-hatching care, whereas females almost never carried out post-laying parental behaviors (i.e., brooding and chick attending). Our findings are consistent with those for other sex-role-reversed shorebirds (e.g., red phalaropes, Tulp et al. 2009) and other jacana species, including Indomalaya-Australasian jacanas (e.g., comb-crested jacanas, Mace 2000) and neotropical jacanas (e.g., northern Jacanas, Betts and Jenni 1991; wattled jacanas *J. jacana*, Emlen and Wrege 2004). In addition, the time allocation varied in both males and females over the breeding stages. The fact that breeding behaviors and foraging dominated the males’ time budget, while females spent most of their time foraging and engaging in territory-related behaviors (i.e., vigilance and preening), suggests that pheasant-tails are so-called “income” breeders (Tulp et al. 2009). Although our study lacks the data of body mass change of the jacanas, this finding suggests that resource availability on sites, such as food (as determined by the diversity and abundance of aquatic plants and invertebrate prey), may play a crucial role in affecting the parents’ contribution (i.e., parental care) to their offspring (e.g., Kentish plovers, Szekély and Cuthill 1999) and subsequently to jacana population.

Our study focused on daytime only, thus we had underestimated the time allocation of incubation and chick attending, which likely happen at night as well. Still, the overall time devoted by males to brooding (53.69% by observation bouts, or 39.84% by individuals) was comparable to that reported elsewhere for pheasant-tailed jacanas (e.g., 56.75%, Fazili et al. 2013) or other jacanas (e.g., 53%, African jacanas *Actophilornis africanus*, Tarboton 1992; 41%, wattled jacanas, Emlen and Wrege 2004). However, strong individual variation was observed. The time devoted by males to chick attending was even lower, less than one-third of the time spent brooding. Nevertheless, this finding along with the observations that the time devoted to chick-attending by males was positively correlated with brood success, while preening was negatively correlated with the fledgling rate, help to explain the low fledging rates (Lee et al. 2024, this study), given the nidifugous nature of the precocious young.

Male pheasant-tailed jacanas generally start brooding after the 2nd or 3rd egg is laid, although they may also do so after the entire clutch is completed (Weng and Wang 1999; Fazili et al. 2013; Chuang 2022). This, together with the intermittent incubating pattern, and in a tropical weather, contributed to an egg mass loss of 17.5 to over 20% during incubation (Fazili et al. 2013, this study), which is comparable to but a little higher than many birds (10–23%, Mueller et al. 2022). Nevertheless, the typical clutches of four eggs (mean mass >50 g) accounted for approximately 22% to over one-fourth of the female body mass of pheasant-tails (ca. 205–250 g; Weng and Wang 1999; Lee et al. 2024) during the prime breeding season. This is substantially higher than that of bronze-winged jacanas (16.8%, Butchart 2000), which are slightly smaller in size but have longer tarsus than pheasant-tails. Furthermore, our data indicate that females maintained this output without reducing the egg size for more than two-third of the breeding season, giving pheasant-tailed jacanas one of the greatest capacities of any shorebird to lay multiple clutches (see Butchart 2000).

While reported variation in avian egg size is mainly inter-clutch rather than intra-clutch (Christians 2002), eggs of pheasant-tailed jacanas in the present study showed intra-clutch variation with the 4th egg being the lightest. This is consistent with and may further enhance the effect of their asynchronous hatching (e.g., Lengyel et al. 2009) as an aid to brood reduction or more adaptively as bet-hedging strategy (Slagsvold et al. 1984; Laaksonen 2004; Fuertes-Recuero et al. 2025). Our result contrasts that of wattled jacanas (Osborne and Bourne 1977; *n* = 1–3), but was based on a much larger sample. Nevertheless, verifying either inference demands data on the fate of individual eggs and post-hatching offspring (see Maddox and Weatherhead 2008), which are lacking in the present study and require future extensive marking and long-term field monitoring.

We also found that the egg mass was greater in later clutches, consistent with that in some other polyandrous shorebirds (e.g., snowy plovers *Charadrius nivosus*, Eberhart-Hertel et al. 2023). In addition, clutches from polyandrous females had greater egg mass. A greater egg mass contributes directly to the offspring quality (i.e., greater sized hatchlings and fledglings; Krist 2011) as well as brood success. Our data, thus, suggest that the individual quality of females in term of egg production may play a crucial role for jacanas in environments facing high predation risks (Butchart 2000; Lee et al. 2024) and the energetic costs of parental care is relatively low. This is further supported by the substantial female investment through their gamete production. From the perspective of investment, this challenges the conventional view that almost only male jacanas care for the young (Cockburn 2006). While males are responsible for the direct care, females invest through egg production and provisioning, and both parents contribute to the fitness of the offspring.

Egg laying represents a significant female input, and may even impose a greater cost on females than rearing offspring (e.g., Heaney and Monaghan 1995; Visser and Lessells 2001). Female jacanas can produce many eggs over multiple clutches, either with multiple or single mates (Betts and Jenni 1991; Emlen and Wrege 2004; Lee et al. 2024), while maintaining a consistent egg size (this study). This capacity of egg-producing must place a substantial demand on the females’ body condition and maintenance, which may contribute to a higher adult female mortality (Romano et al. 2022) and shortened lifespan of females (Tarboton 1992). The relevant data of pheasant-tailed jacanas request further studies; however, black coucals in the field (Goymann et al. 2015) and barred buttonquails (Leitner et al. 2021) in captivity have confirmed this speculation. Without deviating from the parity of hatchling sex ratio (reviewed in Leitner et al. 2021), this inference provides an additional but not mutually exclusive alternative to the female-biased mortality (e.g., red-necked phalarope, English et al. 2014; snowy plovers, Eberhart-Phillips et al. 2017). The female-biased mortality in turn facilitates a male-biased adult sex ratio (ASR; Szekély et al. 2014a) that may reinforce the maintenance of polyandry or contribute to the emergence and evolution of sex role reversal, not only in birds (Liker et al. 2013, 2015; Szekély et al. 2014b; Safari and Goymann 2021; Fresneau et al. 2024) but also in honey locust beetles (*Megabruchidius dorsalis*, Fritzsche et al. 2016) and other taxa (reviewed in Gwynne 1991; Fritzsche et al. 2021).

## Data Availability

Please contact the corresponding author.
